# A Simple Method for Simulating Drought Effects on Plants

**DOI:** 10.3389/fpls.2019.01715

**Published:** 2020-01-21

**Authors:** Renée M. Marchin, Alessandro Ossola, Michelle R. Leishman, David S. Ellsworth

**Affiliations:** ^1^ Hawkesbury Institute for the Environment, Western Sydney University, Penrith, NSW, Australia; ^2^ Department of Biological Sciences, Macquarie University, North Ryde, NSW, Australia

**Keywords:** glasshouse experiment, moderate drought stress, plant drought tolerance, plant ecophysiology, soil water content, water deficit

## Abstract

Drought is expected to increase in frequency and severity in many regions in the future, so it is important to improve our understanding of how drought affects plant functional traits and ecological interactions. Imposing experimental water deficits is key to gaining this understanding, but has been hindered by logistic difficulties in maintaining consistently low water availability for plants. Here, we describe a simple method for applying soil water deficits to potted plants in glasshouse experiments. We modified an existing method (the “Snow and Tingey system”) in order to apply a gradual, moderate water deficit to 50 plant species of different life forms (grasses, vines, shrubs, trees). The method requires less maintenance and manual handling compared to other water deficit methods, so it can be used for extended periods of time and is relatively inexpensive to implement. With only a few modifications, it is possible to easily establish and maintain soil water deficits of differing intensity and duration, as well as to incorporate interacting stress factors. We tested this method by measuring physiological responses to an applied water deficit in a subset of 11 tree/shrub species with a wide range of drought tolerances and water-use strategies. For this subgroup of species, stomatal conductance was 2–17 times lower in droughted plants than controls, although only half of the species (5 out of 11) experienced midday leaf water potentials that exceeded their turgor loss (i.e., wilting) point. Leaf temperatures were up to 8°C higher in droughted plants than controls, indicating that droughted plants are at greater risk of thermal damage, relative to unstressed plants. The largest leaf temperature differences (between droughted and well-watered plants) were in species with high rates of water loss. Rapid osmotic adjustment was observed in leaves of five species when drought stress was combined with an experimental heatwave. These results highlight the potential value of further ecological and physiological experiments utilizing this simple water deficit method to study plant responses to drought stress.

## Introduction

Drought has been described as the most damaging climate hazard facing our global population (Kogan, 1997). It is expected to increase in frequency and severity in many regions in the future as a result of decreased precipitation and increased evaporation due to global climate change (IPCC, 2018; Naumann et al., 2018; Dey et al., 2019). The spatial extent and duration of recent droughts, such as the Millennium Drought (1997–2009) in southern Australia and the California drought (2011–2017) in the USA, are without precedent within at least the last 400 years (Griffin and Anchukaitis, 2014; Freund et al., 2017). Approximately two-thirds of the global population will be affected by increasing drought (Naumann et al., 2018), which threatens food security (FAO, 2018), forest health (Allen et al., 2010; Choat et al., 2018), and even the global beer supply (Xie et al., 2018). One of the key challenges for plant science is to improve our understanding of how drought affects plant ecology and plant functional traits, as this will impact agricultural productivity as well as vegetation management. One invaluable and long-used method for examining plant drought responses is the experimental application of controlled water deficits in the glasshouse.

Methods for applying soil water deficits in pot studies date back at least 50 years, but there has not been consensus on a best-practice method (Munns et al., 2010). The most basic method for generating soil water deficits is passive pot-drying by withholding irrigation, but this method risks fast drying rates that do not adequately mimic natural soil water deficits (Poorter et al., 2012). Many early studies added osmotically-active substances (e.g., polyethylene glycol, PEG) to soil (Zur, 1966), but PEG can limit oxygen diffusion to roots (Mexal et al., 1975) and interfere with ion uptake (Yeo and Flowers, 1984). Various other methods have been used to decrease water availability to plants, including reduction of water pressure inside microporous tubes (Steinberg and Henninger, 1997) or attachment of a vacuum pump to pots (Bunce and Nasyrov, 2012), both of which require an additional apparatus and complex logistics to implement. The most commonly used method for applying soil water deficits is to air-dry and regularly weigh individual pots, adding precise amounts of water in order to balance water loss from transpiration and establish the target soil water content (e.g., Earl, 2003). This method successfully simulates drought stress for plants of different sizes, but requires a complex and expensive automated computer system. While the same method can be achieved manually, it is a laborious and time-consuming task, particularly for large experiments with hundreds of plants. Large experimental designs usually require the use of several glasshouses and/or facilities, thus increasing the need for a simple yet accurate method to simulate drought.

**Figure 1:**
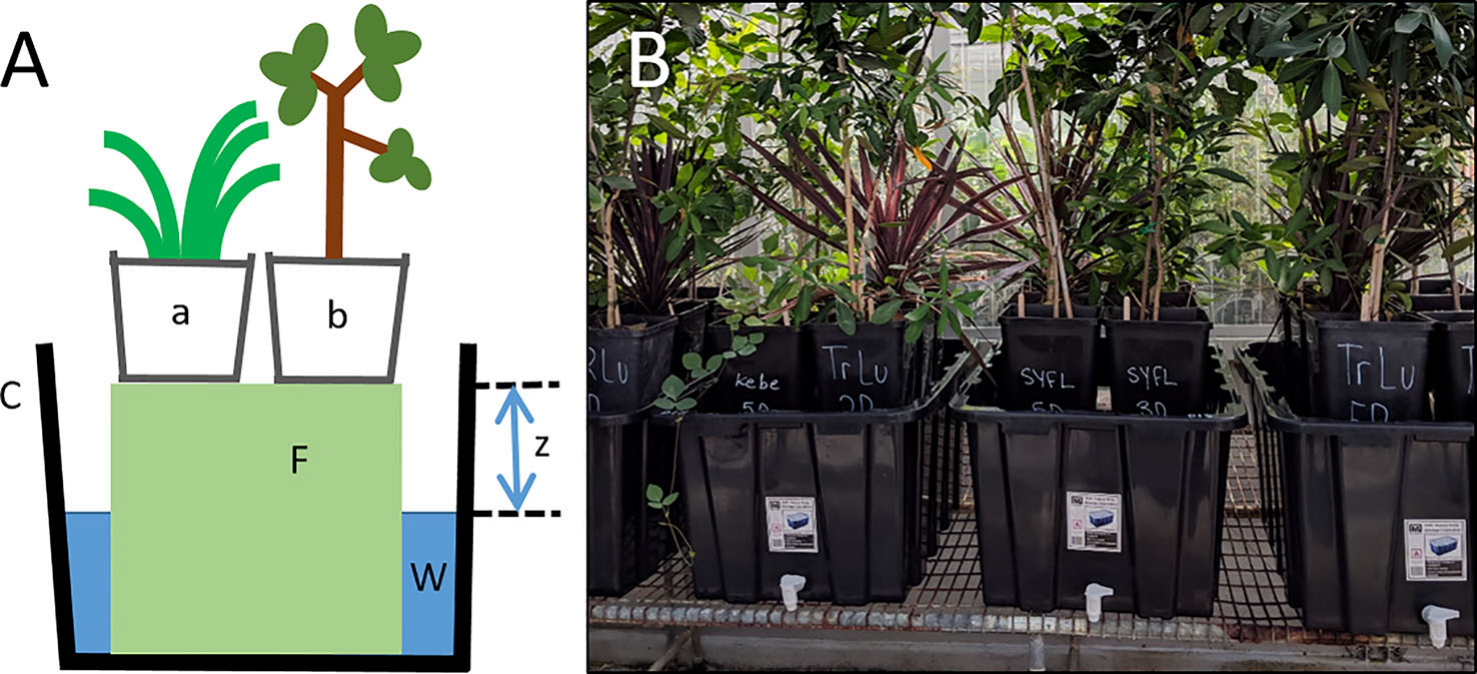
**(A)** Diagram of the simple water deficit method, adapted by permission from Springer Nature Customer Service Centre GmbH: Springer-Verlag *Oecologia* (Potential growth and drought tolerance of eight desert grasses: lack of a trade-off?, Fernández and Reynolds), ^©^ 2000. Capillary irrigation is used to control soil water content of potted plants (a, b), which are placed above a solid column of low water permeability (F, commercial porous foam) inside a plastic container (C) filled with water (W). Intensity of the water deficit is controlled by the depth to water table (z). **(B)** Photo of drought tubs inside the MQ glasshouse.

**Figure 2:**
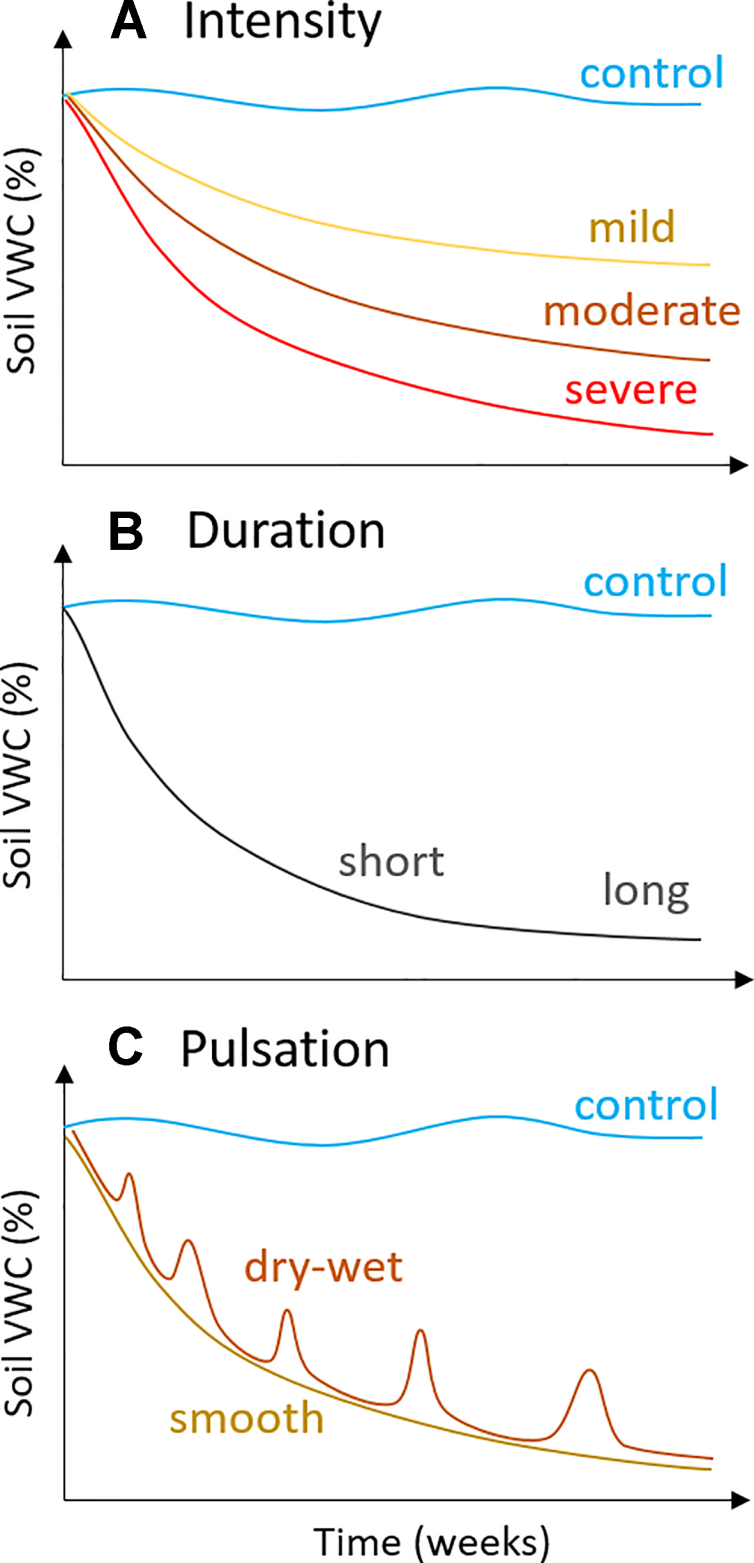
Potential drought scenarios, i.e., experimental water deficit treatments, that can be achieved using the modified “Snow & Tingey system”: **(A)** intensity, from mild to severe, which can be achieved using different depths to water table (z in **Figure 1**: mild, z ≤10 cm; severe, z ≥25 cm), **(B)** duration, from weeks to months, and **(C)** pulsation, i.e., the use of repeated drying and wetting cycles, which can be achieved through repeated lowering and raising of the depth to water table (z in **Figure 1**).

One method that has received relatively little attention was first proposed by Haan and Barfield (1971) and later described by Snow and Tingey (1985), where solid columns of low water permeability are used to separate the root zone from a water table. The original method utilized complex float chambers to establish a more constant water stress (Snow and Tingey, 1985), relative to methods involving repeated cycles of rewatering, and has since been modified several times (Wookey et al., 1991; Fernández and Reynolds, 2000). A simple and inexpensive modification uses commercial floral foam as the means for establishing soil water deficit of potted plants (Fernández and Reynolds, 2000; **Figure 1**). Some other advantages of this version of the ‘Snow and Tingey system’ are: (1) very little maintenance is required, (2) it can be used to apply water deficits simultaneously to diverse plant species with different growth forms, growth rates, sizes, and leaf areas, and (3) it is simple to establish water deficits of varying intensity, duration, and pulsation (i.e., repeated drying and wetting cycles; **Figure 2**). This method is especially useful for establishing gradual water deficits lasting for weeks/months and incorporating interacting stress factors. Previous experiments used pure sand as the potting media (Fernández and Reynolds, 2000; Maseda and Fernández, 2016), which has likely limited the use of this method to date. Here, we revisit the “Snow and Tingey system” and adapt it to simulate a moderate drought stress for a diverse set of plants with different life forms (grasses, vines, shrubs, trees), extending its use beyond sand-based media to allow the use of more complex horticultural media and soils.

Drought usually takes months or years to develop in natural ecosystems (Zargar et al., 2011). Although pot studies cannot recreate the complex heterogeneous factors present in field environments, it is good practice to establish a gradual intensification of water deficit over at least several weeks (Snow and Tingey, 1985). Partial stomatal closure is one of the earliest responses to water deficit and can occur rapidly (i.e., within days), but acclimation responses – such as osmotic adjustment – require longer stress events (Harb et al., 2010; Blum, 2017). Osmotic adjustment is the accumulation of solutes in plant leaves under water deficit and is a strategy for maintaining turgor at low leaf water potentials (Hsiao et al., 1976; Morgan, 1984; Chen and Jiang, 2010). Plant species vary in their ability to osmotically adjust (Bartlett et al., 2014), and the adaptive process of osmotic adjustment requires time, at least 6–15 days of water deficit in crop varieties with the capacity for high osmotic adjustment (Blum et al., 1980; Molinari et al., 2004; Blum, 2017). Other drought responses, such as wilting and loss of stem conductivity, require more severe drought stress (Bartlett et al., 2016). Damage to the light-harvesting capacity of leaf photosynthesis occurs only after severe loss of hydraulic function under extreme dehydration (Trueba et al., 2019). In this way, it is important to carefully consider the desired level of drought response before selecting an experimental water deficit treatment (see **Figure 2**).

We designed glasshouse experiments to screen large numbers of plant species (>50 species) in order to identify drought-tolerant species for urban plantings in Australian cities, though the drought method we describe can be used in a wide variety of contexts. Drought is common in Australia (Nicholls et al., 1997; Freund et al., 2017) and has been associated with tree decline in urban areas (Nitschke et al., 2017). Unprecedented extreme temperatures are also predicted for many parts of the world, including Australia, within the next 10–30 years (Perkins and Alexander, 2013; Lewis et al., 2017; BoM, 2019), so we tested the efficacy of this method by applying an experimental heatwave in the fifth week of water deficit to better understand how plants will cope with combined drought and heat stress. The overall goals of this work were to: (1) refine a simple water deficit method to simulate drought stress on potted plants, (2) evaluate its effectiveness in generating some common ecophysiological responses to water deficits, and (3) demonstrate that this method can be used to investigate interactive effects of drought and other environmental stresses, such as heatwaves. We show how the gradual, moderate water deficit achieved with the “Snow and Tingey system” affected plant physiological responses in a subset of 11 tree/shrub species. We also discuss the versatility of this simple method for simulating drought effects on plants in order to highlight potential uses in other ecological experiments.

## Materials and Methods

### Plant Material and Experimental Conditions

Two coordinated glasshouse experiments were conducted: one at the Hawkesbury Institute for the Environment (HIE; Western Sydney University, Richmond, NSW, Australia) and the other at Macquarie University (MQ; North Ryde, NSW, Australia). A total of 50 plant species from 20 different families were selected, including 25 tree, 11 shrub, 7 vine/groundcover, and 7 herbaceous species (**Supplementary Table S1**). Species included both native Australian plants (42 species) and exotic species (8 species) and occur in a range of different environments, from semi-arid to rainforest ecosystems (**Table 1**). Twenty-four species were grown in two glasshouse bays at HIE from 1 November 2017 to 23 March 2018; 26 species were grown in two glasshouse bays at MQ from 26 January to 25 May 2018. Planting stock (*n* = 10 plants per species) was obtained from commercial nurseries in Australia as tubestock, 140-mm, or 200-mm pot size, depending on species availability. Seedlings were bare-rooted and transplanted into 6-L square pots containing native potting mix (<30% sand/coir, >70% screened composted pine bark; Australian Growing Solutions, Tyabb, VIC, Australia), 38 g of controlled-release native plant fertilizer (Scotts Australia Osmocote Slow Release, Bella Vista, NSW, Australia), and a 1.25-g tablet of systemic insecticide and fertilizer (Yates Confidor, Padstow, NSW, Australia).

**Table 1:** Eleven tree/shrub species selected for detailed leaf-level physiological measurements, ranked from low to high drought tolerance using mean water potential (± SE) at turgor loss point (π_tlp_, MPa) estimated from osmometer measurements of π_o_ (*n =* 16–20 leaves per species). Species abbreviation (used in **Figures 6**–**8**) and natural occurrence are also provided, as well as minimum midday water potential (Ψ_mid_, MPa) during the heatwave and mean wood density (± SE, g cm^-3^; *n* = 4–5 plants per species).

Species	Species Code	Natural Occurrence	π_tlp_ (MPa)	Minimum HW Ψ_mid_ (MPa)	Wood density (g cm^-3^)
*Magnolia grandiflora* L.	Magr	warm temperate forest	-1.71 ± 0.02	-1.85	0.46 ± 0.01
*Castanospermum australe* A.Cunn. & C.Fraser	Caau	tropical rainforest	-1.71 ± 0.03	-1.73	0.50 ± 0.02
*Lophostemon confertus* (R.Br.) Peter G.Wilson & J.T.Waterh.	Loco	sclerophyll forest, rainforest	-1.71 ± 0.03	-2.65	0.42 ± 0.01
*Syzygium floribundum* F.Muell.	Syfl	riverine rainforest	-1.78 ± 0.04	-2.00	0.56 ± 0.04
*Hymenosporum flavum* F.Muell.	Hyfl	sclerophyll forest, rainforest	-1.94 ± 0.03	-3.06	0.59 ± 0.01
*Toechima erythrocarpum* (F.Muell.) Radlk.	Toer	tropical rainforest	-1.99 ± 0.06	-2.45	0.64 ± 0.03
*Harpullia pendula* Planch. ex F.Muell.	Hape	subtropical to tropical rainforest	-2.05 ± 0.05	-2.65	0.66 ± 0.03
*Hakea laurina* R.Br.	Hala	semi-arid mallee plain	-2.08 ± 0.03	-2.56	0.44 ± 0.02
*Hakea salicifolia* (Vent.) B.L.Burtt	Hasa	sclerophyll forest	-2.12 ± 0.04	-3.85	0.49 ± 0.004
*Grevillea baileyana* McGill.	Grba	tropical rainforest	-2.21 ± 0.02	-2.98	0.53 ± 0.01
*Buckinghamia celsissima* F.Muell.	Buce	tropical rainforest	-2.30 ± 0.02	-2.75	0.55 ± 0.01

For additional species information (horticultural variety, family, growth form, origin), see **Supplementary Table S1**.

All seedlings were well-watered using drip irrigation for 6–15 weeks to establish roots, allow formation of new leaves, and acclimate to glasshouse environmental conditions. During this time, seedlings were rotated within and between glasshouse bays on a monthly basis to allow uniform solar irradiance for growth. At the beginning of the experiment, seedlings received one daily watering of 1 L at 6:00. As seedlings grew, watering was increased to a total of 1.5–4.5 L daily (delivered at 8:00, 13:00, and 17:00) to keep all seedlings well-watered. The average glasshouse temperature was 27°C to represent summer conditions in southeastern Australia, with a diurnal range from 21 to 34°C and the maximum temperature spanning 6 h at midday (12:00–18:00; **Supplementary Figure S1**). Glasshouse daytime relative humidity ranged from 40%–95%, which led to a range in daytime vapor pressure deficit (VPD) of 0.2–3.5 kPa. Daily maximum photosynthetically active radiation (PAR) was >2,000 µmol m^-2^ s^-1^ inside the HIE glasshouses, but only ~1600 µmol m^-2^ s^-1^ inside the MQ glasshouses.

### Water Deficit Treatment

After the acclimation period, half of the plants (*n =* 5 plants per species) were exposed to a gradual, five-week water deficit using the method described by Snow and Tingey (1985) and modified by Fernández and Reynolds (2000). Pure sand has been used to achieve fast equilibrium rates during drying (Fernández and Reynolds, 2000), but it is not a preferred growth substrate for most plants. We used a native potting mix (as described above; bulk density: 0.45 ± 0.004 g cm^-3^), instead of pure sand, to successfully grow a diverse set of 50 plant species (**Supplementary Table S1**). It was not possible to drought all species in the same week due to a limited number of glasshouses, so 5–6 species with similar growth rates were batched and treated at the same time.

Before planting, four large 4.3-cm diameter circles were drilled into the flat base of each drought pot and fitted with fine nylon mesh (20-µm, Allied Filter Fabrics, Berkeley Vale, NSW) to allow exchange of air and water but prevent root passage (**Supplementary Figure S2**). Utility taps were installed into the base of 100-L plastic containers (hereafter referred to as drought tubs) to allow for water drainage. Pots were placed inside drought tubs on top of a 23-cm column of commercial porous foam (Oasis IDEAL Floral Foam Maxlife brick; Smithers-Oasis, Kent, OH, USA) with an adjustable water table. Pots were able to be removed as needed for measurements, and the constant water deficit was restored after pots were replaced onto the commercial foam. However, it is essential to this capillary irrigation method that: (1) there is adequate contact area between the soil at the bottom of the pot and the foam surface and (2) the pore size of the commercial foam is sufficient for transporting water to the desired height by capillarity.

Depth to the water table was progressively increased every day based on a predetermined schedule designed for the soil type, from 1 cm (on day 1) to 15 cm (on day 8) to 22 cm (on day 15), where it was maintained for an additional three weeks (until day 35). Water levels were checked daily and maintained within ±1 cm of the target level, but this rate of change in the water table matched changes in plant transpiration closely so that very little maintenance was required over the first 15 days of drought. The soil volumetric water content (VWC) of each drought pot was measured weekly using a 20-cm water content probe (CS658 HydroSenseII, Campbell Scientific Inc., Logan, UT, USA) for the first three weeks of water deficit, but every 3–4 days thereafter to ensure the final target intensity was reached. The target drought intensity was a soil VWC of 7.5 ± 2.5%, which is below the permanent wilting point for this soil (14%, **Supplementary Figure S3**). Soil VWC was always measured in the morning (8:00–10:00). Pots exceeding the upper limit of our target intensity on day 22 (>10%) were bench-dried to achieve the target intensity, then replaced in the drought tub. This only occurred in a limited number of pots containing small plants with low transpiration rates (e.g., *Cryptocarya laevigata*, *Lophostemon confertus*, *Myoporum parvifolium*). If soil VWC surpassed the lower limit of our target intensity (<5%), 300 ml water was added to the soil surface of the pot to maintain the target soil VWC. This was necessary for pots containing large plants with high transpiration rates (e.g., *Hakea laurina*, *Murraya paniculata*, *Stenotaphrum secundatum*). Thus, additional maintenance was required during the final two weeks of water deficit (days 25–35), although the total time investment was still considerably less than for some other water deficit methods.

All control pots (*n* = 5 plants per species) were maintained at field capacity (soil VWC ~35%, ranging from 25%–45%) for the duration of the experiment. To determine if drought tubs were necessary for watering control plants, we first tested if soil VWC of control pots differed between our two watering methods: capillary irrigation versus drip irrigation. We drilled four 4.3-cm diameter circles into the flat base of 14 control pots, fitted with fine nylon mesh (before potting), and placed pots inside 100-L plastic containers on top of a 23-cm column of commercial foam with a constant 1–4 cm depth to the water table. The soil VWC of each control pot was measured weekly using a 20-cm soil water content probe (CS658 HydroSenseII, Campbell Scientific Inc.). For a subset of four pots, we continuously measured soil VWC using soil water content probes (ThetaProbe type ML2X, Delta-T, Cambridge, UK) at a 10-cm depth to compare: (1) the two methods for watering control pots, and (2) soil VWC differences between a control and drought pot. Output from these soil water content probes was converted into soil VWC using generalised settings for organic soil, but further normalization was required to match point measurements of soil VWC. A constant offset (10%–20%) was applied to the data from each probe to achieve consistency across all soil VWC measurements.

In the final week of water deficit (days 29–35), we applied an experimental heatwave to the droughted plants to better understand how plants cope with combined stress factors. We exposed droughted plants to a 7-day heatwave by moving pots into another glasshouse chamber maintained at higher temperatures than the growth regime. The average heatwave temperature was 35°C, with a diurnal range of 30°C–41°C and the maximum temperature spanning 2 h at midday (12:00–14:00; **Supplementary Figure S1**).

### Pressure-Volume Curves and Leaf Osmotic Potential

Pressure-volume curves were measured for well-watered plants of 29 species (*n* = 1–10 leaves per species) to allow estimation of the water potential at wilting point (or turgor loss point, π_tlp_), which is strongly related to plant drought tolerance (Engelbrecht et al., 2000; Baltzer et al., 2008). Logistical constraints prevented measurement of all 50 species. The pressure-volume curves were measured using a pressure chamber (Model 1505D, PMS Instrument Company, Albany, OR, USA) following the bench-drying method (Tyree and Hammel, 1972; Schulte and Hinckley, 1985). Leaves were rehydrated overnight for 12 h using the standing rehydration method (Arndt et al., 2015) to ensure leaves were fully hydrated. Leaf fresh weight (g) and leaf water potential (Ψ_leaf_, MPa) were measured periodically as leaves dried under ambient laboratory conditions. Leaf dry mass was measured after oven-drying at 70°C for 72 h. We determined π_tlp_, osmotic potential at full turgor (π_o_), relative water content at turgor loss point (RWC_tlp_), and bulk modulus of elasticity (ε) following standard methods (Turner, 1981) using a pressure-volume curve analysis routine developed by Kevin Tu (available at: http://landflux.org/Tools.php, accessed 1 November 2019) and based on Schulte and Hinckley (1985).

The π_o_ of fully-expanded, fully-hydrated leaves was also measured independently using an osmometer (WP4C Dewpoint PotentiaMeter, Decagon Devices, Pullman, WA, USA) for these 29 species (*n =* 5–10 leaves per species), following the method described by Bartlett et al. (2012). Briefly, leaves and/or stems were collected and rehydrated overnight for 12 h using the standing rehydration method (Arndt et al., 2015). Rehydration may cause solute leakage into the apoplast and underestimation of osmotic potential in some plant species (Kubiske and Abrams, 1991; Arndt et al., 2015), but was necessary to ensure fully-hydrated leaves (i.e., Ψ_leaf_ ≥ –0.3 MPa) were used for comparison of control and droughted plants. The midrib was removed from leaves before 40-mm diameter leaf discs or leaf pieces were quickly cut from each plant. Leaf pieces were wrapped in foil and frozen in liquid N_2_ for 2 min, then equilibrated for 10 min inside a sealed, humidified plastic bag. Leaf pieces were punctured repeatedly with sharp-tipped forceps immediately before measurement using the osmometer. Measurements were recorded for 20–30 min, until equilibrium as indicated by <0.01 MPa change over 2 min. Osmometer measurements of π_o_ were used to estimate species π_tlp_ using the following equation: π_tlp_ = 0.832π_o_ – 0.631 (Bartlett et al., 2012).

### Plant Physiological Responses to Water Deficit Treatment

Eleven of the 50 species, all broadleaf evergreen trees/shrubs (**Table 1**), were selected to assess the impact of the soil water deficit on plant function. We collected a set of leaf-level physiological measurements: stomatal conductance (g_s_), leaf water potential (Ψ_pre_, Ψ_mid_), leaf temperature (T_leaf_), and leaf osmotic potential (π_o_). Control and drought plants (*n =* 4–5 plants per treatment) were measured under the target soil VWC (34%–41% vs. 5%–11%, respectively) during the fourth week of water deficit.

Stomatal conductance was measured on sunny days at midday (11:00–14:00) on three fully-expanded leaves per plant using a porometer (AP-4, Delta-T, Cambridge, UK). The same leaves (*n =* 1–2 leaves per plant) were subsequently removed for measurement of Ψ_mid_ with a pressure chamber (Model 1505D, PMS Instruments). Measurements of g_s_ and Ψ_mid_ were repeated on two different days and averaged for each plant. Leaves (*n =* 1–2 leaves per plant) for measurement of Ψ_pre_ were collected at 5:00. All leaves for water potential measurement were stored inside a sealed, humidified plastic bag and kept cool and dark until measurement, which was completed within 3 h of collection.

Leaf temperature was measured on sunny days (9:00–14:00) on three fully-developed leaves per plant using an infrared thermometer (Agri-Therm III Model 6110L, Everest Interscience, Inc., Chino Hills, OR, USA) held at a distance of about 10 cm from the leaf surface. Thermal emissivity was set to 0.92, a representative value for individual plant leaves (Jones, 2004).

The π_o_ was measured in both the fourth and fifth weeks (on the fifth heatwave day) of drought using an osmometer (WP4C Dewpoint PotentiaMeter, Decagon Devices) following the Bartlett et al. (2012) method described above. Leaves (*n =* 1–2 leaves per plant) were also collected on the fifth heatwave day for measurement of Ψ_mid_ with a pressure chamber (Model 1505D, PMS Instruments). After the end of the heatwave, all plants (including drought plants) were well-watered then monitored for survival after a two-week recovery period.

Wood density was determined for the 11 focal species (*n =* 4–5 plants per species) after the completion of the experiment. A 5-cm stem segment was split to remove the pith and bark before determining fresh volume using the water displacement method. The wood sample was then dried to constant mass at 70°C and weighed.

### Statistical Analyses

Differences in soil VWC between control and drought pots were analyzed in the fourth week of drought by using a Student’s *t* test. The overall effect of drought on g_s_, T_leaf_, Ψ_pre_, Ψ_mid_, and π_o_ was determined by using full‐factorial, mixed‐model analyses of variance (ANOVAs) with species and treatment as the main effects; species was analyzed as a random effect, and treatment was analyzed as a fixed effect. When there was a significant species × treatment interaction (*p* ≤ 0.05), individual species’ responses were analyzed using Student’s *t* tests. Separate paired-sample Student’s *t* tests were used for each species to analyze differences in π_o_ between the fourth and fifth week of drought; values were paired by plant. We used ordinary least squares regression to correlate π_o_ with π_tlp_, then used analysis of covariance (ANCOVA) to test if the slope and intercept of our relationship differed from a previously published relationship by Bartlett et al. (2012). All data were tested for normality with the Shapiro and Wilk’s test; g_s_, T_leaf_, Ψ_pre_, and π_o_ measurements were ln-transformed to achieve normality. All statistical analyses were completed using R Statistical Software 3.5.1 (R Core Team, 2018). Means were considered significantly different at *p* ≤ 0.05.

## Results

### Achieving Experimental Plant Water Deficits

**Figure 3:**
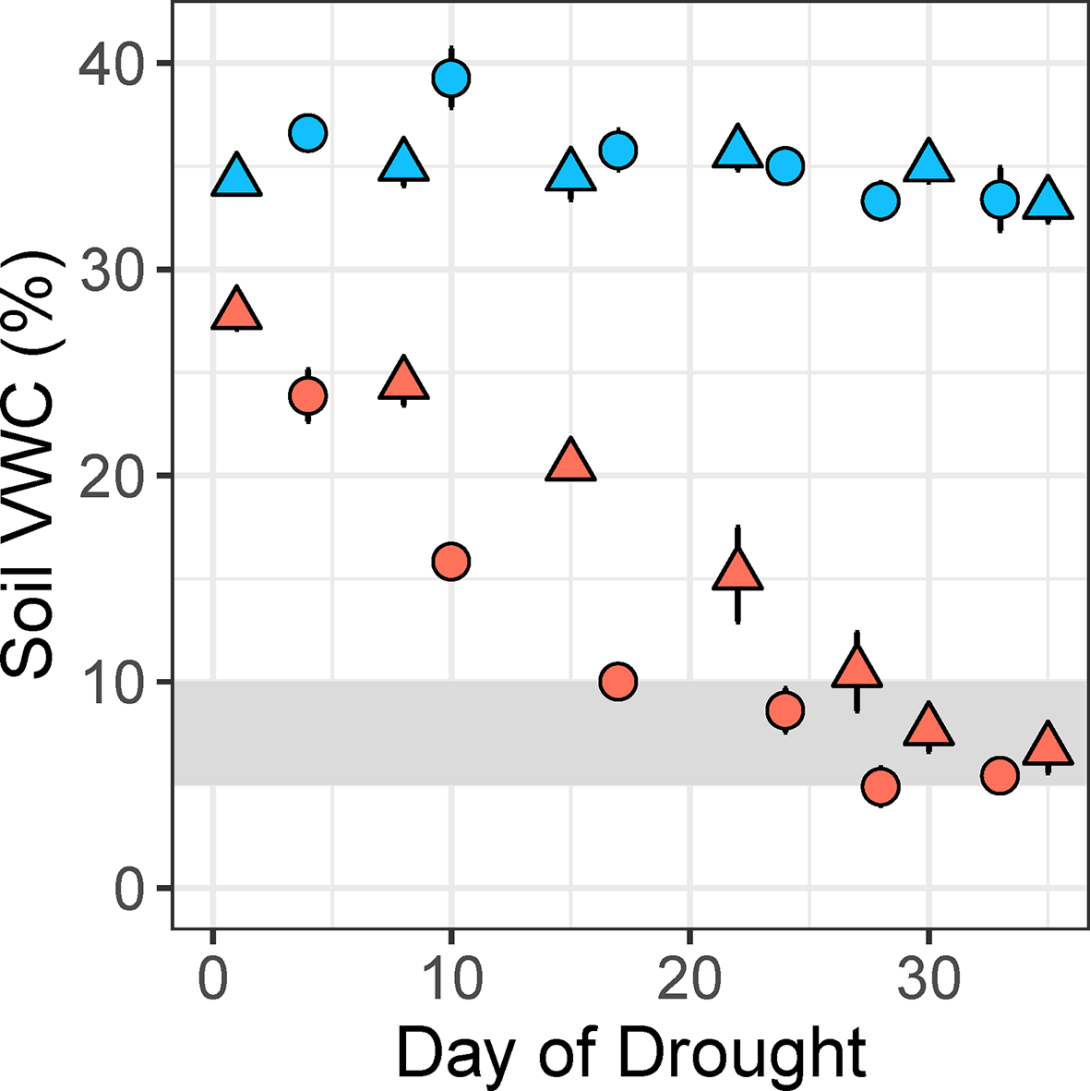
Differences in soil volumetric water content (VWC, %) between control (blue) and drought (coral) pots for two different plant species over the 5-week experimental drought period. The fastest-drying species (*Lomandra longifolia*, circles) and slowest-drying species (*Tristaniopsis laurina*, triangles) reached the target soil VWC of 10% in 17 and 28 days, respectively, in the HIE glasshouse experiment. Values are means of 5 plants per treatment, and error bars indicate SE. The target drought intensity is shaded gray.

**Figure 4:**
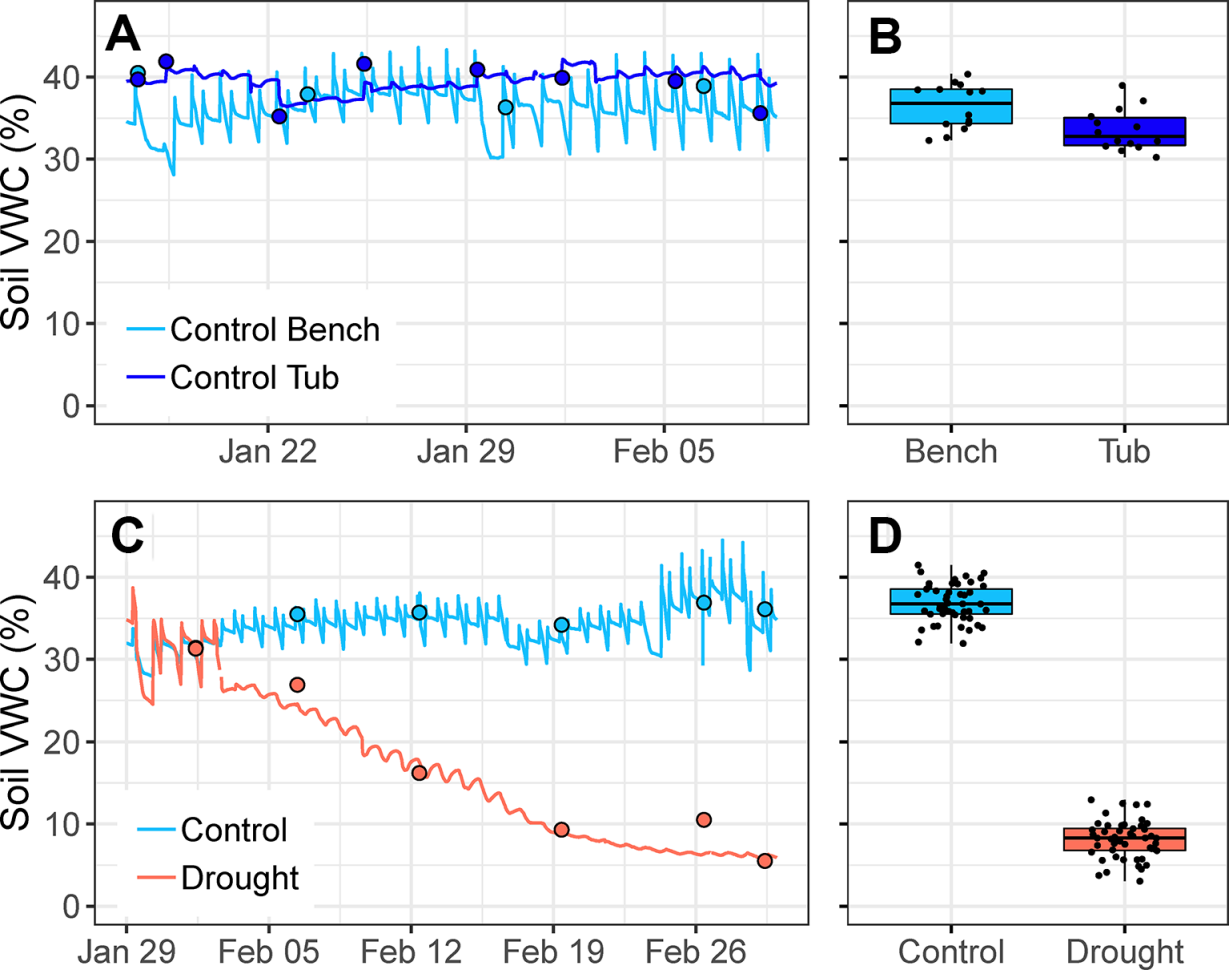
**(A)** Changes in soil volumetric water content (VWC, %) over a 3-week period in the austral summer for one control pot watered *via* drip irrigation on the bench and one control pot watered *via* capillary irrigation (i.e., in plastic tub with water level kept constantly at 1-cm depth). Circles indicate point measurements of soil VWC. **(B)** Mean soil VWC of 14 control pots watered *via* drip irrigation and 14 control pots (of the same species) watered *via* capillary irrigation over a four-week period; treatments are significantly different (*t*_26_ = 2.842, *p* = 0.009). **(C)** Changes in soil VWC over the first four weeks of drought for one control pot (watered *via* drip irrigation) and one drought pot (watered *via* capillary irrigation). From 29 January to 2 February, both pots were well-watered using drip irrigation. Circles indicate point measurements of soil VWC. **(D)** Species mean soil VWC of control and drought treatments for all 50 species during the fourth week of drought; treatments are significantly different (*t*_48_ = 33.275, *p* < 0.001). Points are means of 4–5 plants per treatment.

The experimental treatment gradually reduced soil volumetric water content of potted plants from field capacity (~35%) to a moderate water deficit (target soil VWC: 7.5 ± 2.5%) over a period of several weeks (**Figure 3**). Our method was successfully implemented at two glasshouse locations for a diverse set of 50 plant species, including grasses, vines, shrubs, and trees (**Supplementary Table S1**). The largest plants with the highest transpiration rates reached a soil VWC of 10% in about 15 days, while the smallest plants required 28 days (**Figure 3**). There was a significant difference in soil VWC between the control and drought treatments (*t*_48_ = 33.28, *p* < 0.001) at maximum drought intensity in the fourth week of water deficit (**Figure 4D**). Mean soil VWC was higher in the control treatment, relative to the drought treatment, for all 50 study species (32%–42% vs. 3%–13%, respectively).

Pots that were watered *via* drip irrigation had larger diurnal variation in soil VWC, relative to pots watered *via* capillary irrigation inside drought tubs (**Figures 4A, C**). Either method, drip irrigation or capillary irrigation, was capable of maintaining soil VWC of control pots within the target range (25%–45%), although soil VWC was significantly higher in pots watered *via* drip irrigation (*t*_26_ = 2.84, *p =* 0.009; **Figure 4B**). Mean soil VWC of 14 drip-irrigated control pots ranged from 32%–40%, while mean soil VWC of 14 capillary-irrigated control pots (of the same species) ranged from 30%–39%.

### Plant Physiological Responses to Water Deficit Treatment

**Figure 5:**
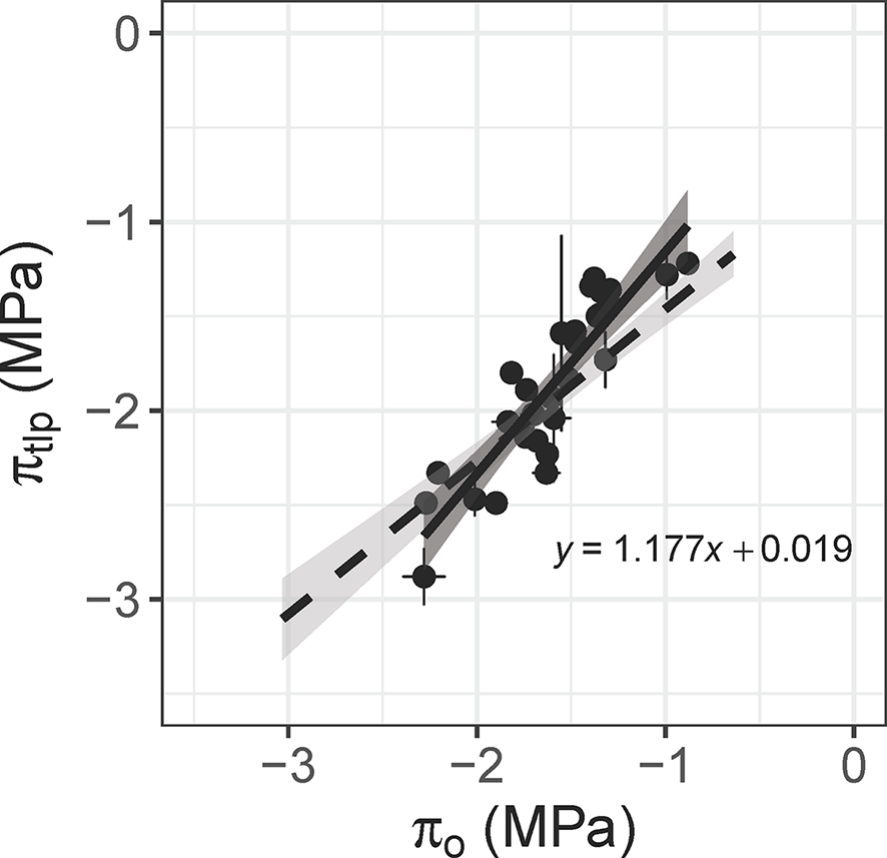
Relationship between leaf osmotic potential (π_o_, MPa) and the water potential at leaf turgor loss point (π_tlp_, MPa) for 29 plant species (*n* = 1–10 leaves per species; *r*^2^ = 0.82, *p* < 0.001). Leaf osmotic potential was measured with an osmometer, and π_tlp_ was calculated from pressure-volume curves. The dashed line is the relationship published by Bartlett et al. (2012), which includes 30 woody species. The slope does not differ between the two relationships within the overlapping range of π_o_ (*F*_1,48_ = 3.181, *p* = 0.081).

Leaf osmotic potential was tightly correlated with π_tlp_ across 29 species/varieties (*r* ^2^ = 0.82, *p* < 0.001; **Figure 5**, **Supplementary Table S2**). This relationship was compared to the relationship published by Bartlett et al. (2012) to verify its use for estimating π_tlp_, and thus drought tolerance, of 11 tree/shrub species used for detailed physiological measurements. The slopes did not differ within the overlapping range of π_o_ (‒0.9 to ‒2.3 MPa; *F*_1,48_ = 3.181, *p =* 0.081), although the intercepts were significantly different (*F*_1,49_ = 6.910, *p =* 0.011). The π_tlp_ ranged from –1.71 to –2.30 MPa and wood density ranged from 0.42‒0.66 g cm^-3^ for these 11 species (**Table 1**), whose natural occurrences span the semi-arid plains to rainforest habitats within Australia. The four species in the Proteaceae family had the lowest π_tlp_ (<–2.0 MPa), indicating high drought tolerance.

**Figure 6:**
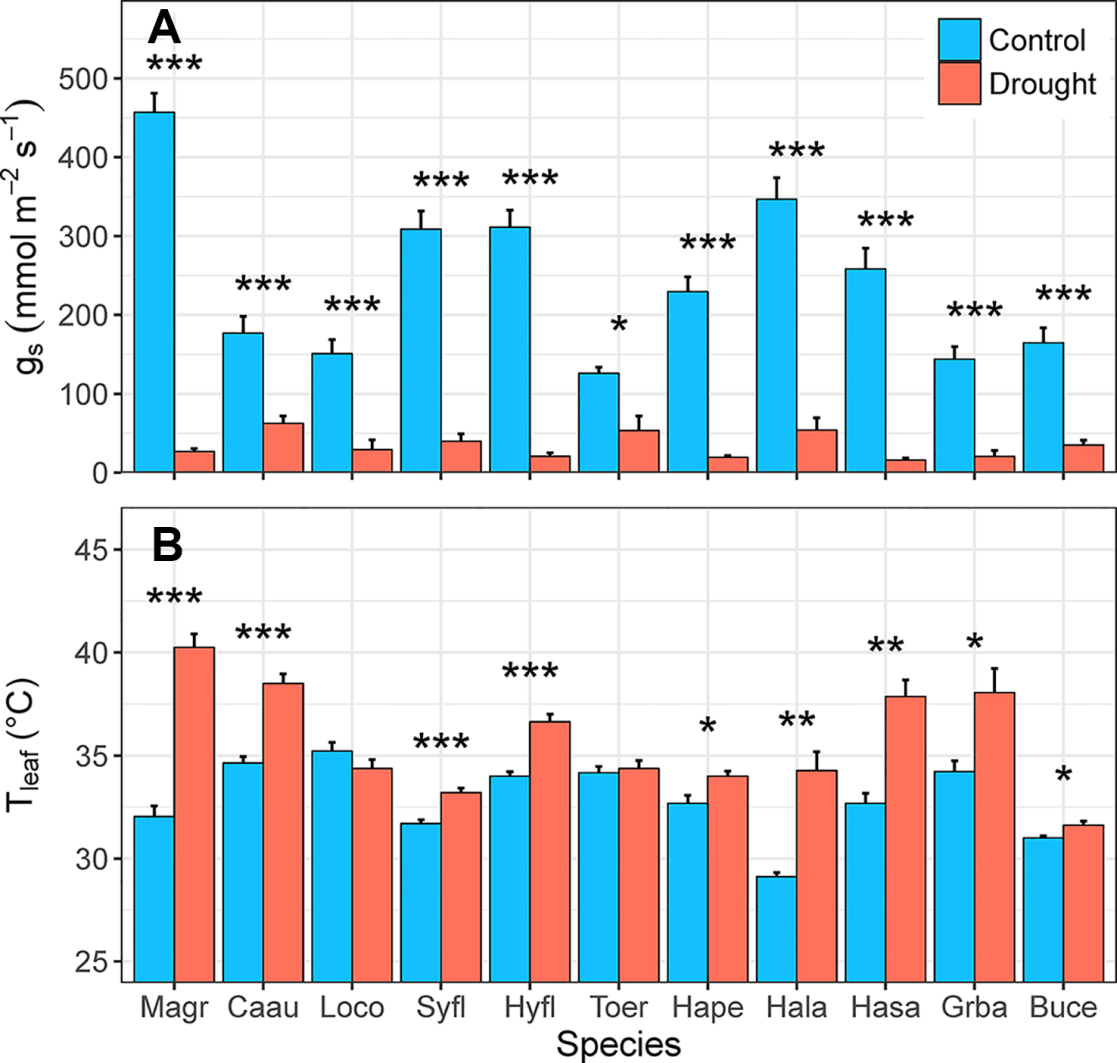
Differences in **(A)** rates of stomatal conductance (g_s_, mmol m^-2^ s^-1^) and **(B)** leaf temperature (T_leaf_, °C) between control and drought plants of 11 selected tree/shrub species. Measurements were completed during the fourth week of drought, when species mean soil volumetric water content (VWC) was 34%–41% in control pots and 5%–11% in drought pots. Species are ordered from low to high drought tolerance according to π_tlp_. Species are denoted according to abbreviations in **Table 1**. Values are means of 4–5 plants, and error bars indicate SE (unidirectional SE are presented for clarity). Asterisks denote significant differences between treatments: *p < 0.05; **p < 0.01; ***p < 0.001.

Droughted plants of all 11 species had significantly lower rates of g_s_, relative to control plants (*F*_1,10_ = 619.29, *p* < 0.001) in the fourth week of water deficit (16–62 vs. 126–457 mmol m^-2^ s^-1^, respectively; **Figure 6A**). For species with high rates of water loss, g_s_ was 6–17 times higher in control plants than in droughted plants. In the species with the lowest rate of water loss (*Toechima erythrocarpum*), g_s_ of control plants was double that of droughted plants. Leaf temperature was also significantly higher for droughted plants, relative to control plants (*F*_1,10_ = 80.177, *p* < 0.001), for 9 of the 11 species (**Figure 6B**). For the species with the highest g_s_ (*Magnolia grandiflora*: 457 mmol m^-2^ s^-1^), T_leaf_ was an average of 8°C higher under simulated drought. The two species for which T_leaf_ did not vary with the water deficit treatment had low rates of g_s_ (*Lophostemon confertus*: 151 mmol m^-2^ s^-1^; *T. erythrocarpum*: 126 mmol m^-2^ s^-1^).

**Figure 7:**
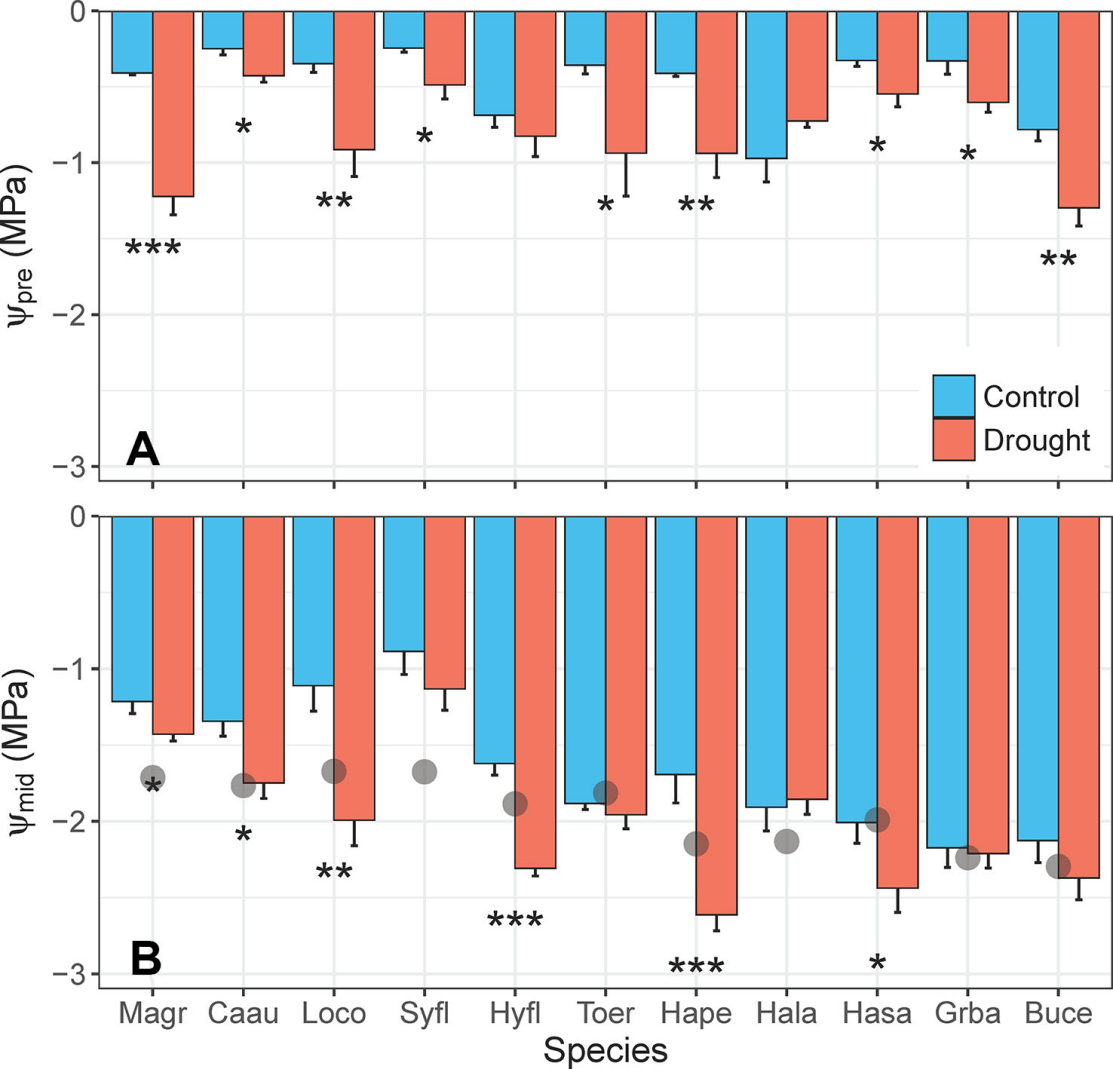
Differences in **(A)** predawn leaf water potential (Ψ_pre_, MPa) and **(B)** midday leaf water potential (Ψ_mid_, MPa) between control and drought plants of 11 tree/shrub species. The gray points in panel **(B)** indicate species mean water potential at leaf turgor loss point (π_tlp_). Measurements were completed during the fourth week of drought, when species mean soil volumetric water content (VWC) was 34%–41% in control pots and 5%–11% in drought pots. Species are ordered from low to high drought tolerance according to π_tlp_. Species are denoted according to abbreviations in **Table 1**. Values are means of 4–5 plants, and error bars indicate SE (unidirectional SE are presented for clarity). Asterisks as in **Figure 6**.

Reductions in Ψ_leaf_ in response to the water deficit treatment were less consistent across species (**Figure 7**). Droughted plants had significantly lower Ψ_pre_ than control plants (*F*_1,10_ = 69.26, *p* < 0.001) for 9 of the 11 species, but all species maintained mean Ψ_pre_ >–1.5 MPa (**Figure 7A**). Only six species had significantly lower Ψ_mid_ in droughted plants, relative to controls (*F*_1,10_ = 33.43, *p* < 0.001; **Figure 7B**). While control plants of all species maintained Ψ_mid_ at or above their turgor loss point, droughted plants of five species had Ψ_mid_ that exceeded their turgor loss point (**Figure 7B**). Severe wilting was observed in *L. confertus* and *Hymenosporum flavum*, but wilting was less obvious for three other species with Ψ_mid_ below their turgor loss point (*T. erythrocarpum*, *Harpullia pendula*, *Hakea salicifolia*).

**Figure 8:**
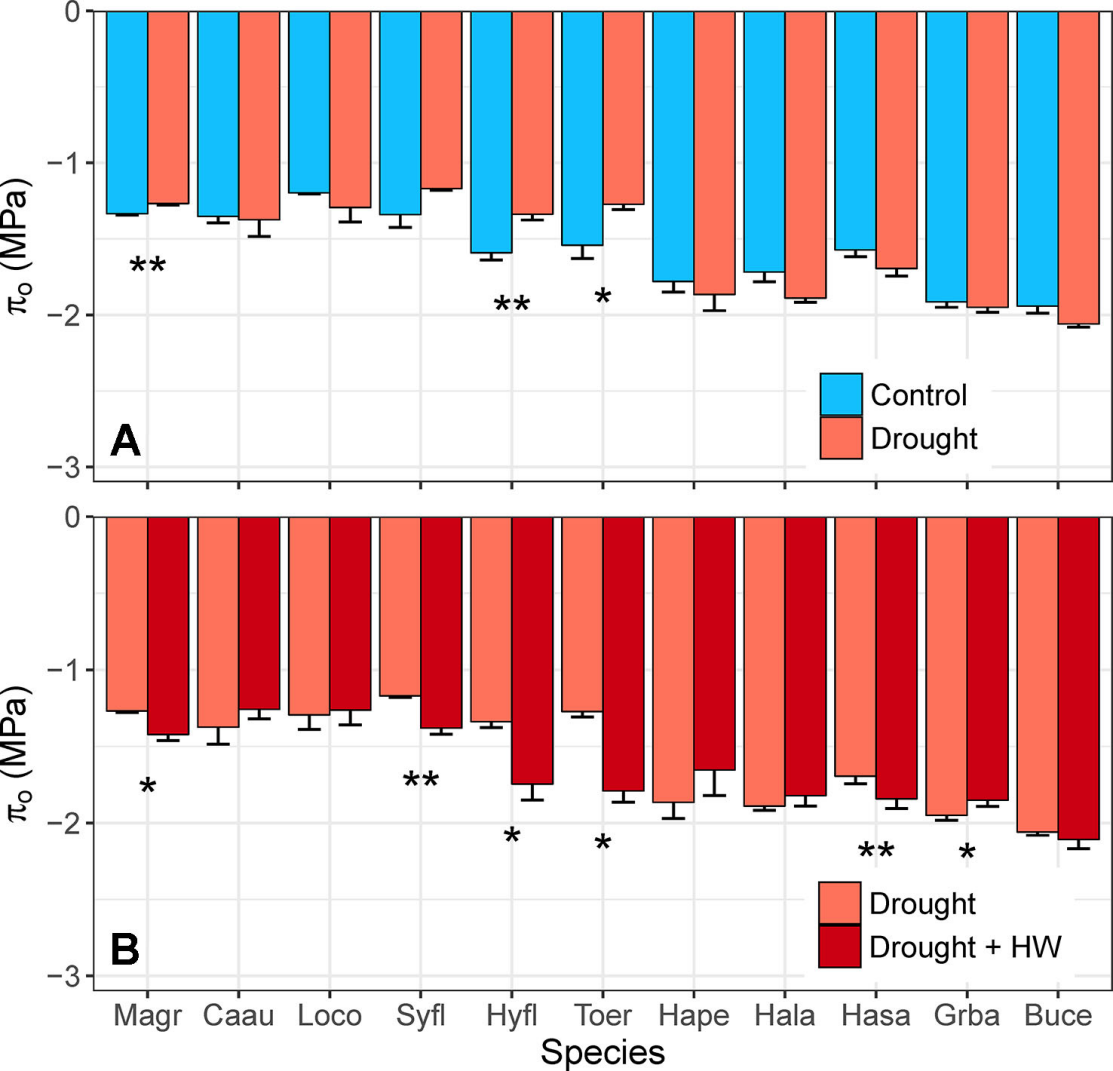
**(A)** Differences in leaf osmotic potential (π_o_, MPa) between control and drought plants of 11 tree/shrub species. Measurements were completed during the fourth week of drought, when species mean soil volumetric water content (VWC) was 34%–41% in control pots and 5%–11% in drought pots. **(B)** Differences in π_o_ of plants between the fourth and fifth week of drought; in week 5, drought plants were exposed to heatwave (HW) temperatures. Species mean soil VWC was 3%–11% in both weeks. Species are ordered from low to high drought tolerance according to π_tlp_. Species are denoted according to abbreviations in **Table 1**. Values are means of 3–5 plants, and error bars indicate SE (unidirectional SE are presented for clarity). Asterisks as in **Figure 6**.

There was no evidence of osmotic adjustment in any species in the fourth week of water deficit (*F*_1,10_ = 1.22, *p =* 0.272). Three species (*M. grandiflora*, *H. flavum*, *T. erythrocarpum*) showed the opposite response, however, by having significantly higher π_o_ in control plants, relative to drought plants (**Figure 8A**). Droughted plants were exposed to heatwave temperatures in the fifth week of drought, which resulted in significant osmotic adjustment (*F*_1,10_ = 4.73, *p* = 0.032) for 5 of the 11 species (**Figure 8B**). For these species, mean π_o_ decreased by –0.15 to –0.54 MPa in just one week. The magnitude of this adjustment surpassed mean π_o_ for control plants of *M. grandiflora*, *H. flavum*, and *T. erythrocarpum* in the fourth week of water deficit. Interestingly, rapid osmotic adjustment was observed for the species with the lowest Ψ_mid_ during the heatwave (‒3.9 MPa, *H. salicifolia*, **Table 1**). Plants of several species showed high levels of leaf desiccation (>50%; *H. salicifolia*, *L. confertus*, *T. erythrocarpum*) as a result of the combination of drought and heat stress, leading to the death of four *H. salicifolia* plants and one *T. erythrocarpum* plant in the following weeks (data not shown).

## Discussion

We modified an experimental method first introduced almost 50 years ago (Haan and Barfield, 1971) to apply a gradual, moderate water deficit (**Figure 3**) to a diverse set of 50 plant species, including grasses, vines, shrubs, and trees (**Supplementary Table S1**). The original method was previously modified to eliminate the need for complex float chambers, but has rarely been used, and only applied to small numbers of species such as desert grasses (Fernández and Reynolds, 2000) and *Eucalyptus* trees (Maseda and Fernández, 2016). Capillary irrigation is used to control the soil water content of potted plants, which are placed above a solid column of low water permeability with an adjustable water table (**Figure 1**, **Supplementary Figure S2**). In these previous experiments, plants were grown in pure sand, which has a relatively high bulk density and low porosity. The use of pure sand can restrict root growth and has likely limited the use of this water deficit method in other studies. Instead of pure sand, we used a potting mix with low bulk density (0.45 ± 0.004 g cm^-3^), which is ideal for root growth as it allows movement of air, water, and nutrients through the soil. The higher water-holding capacity of the potting mix, relative to pure sand, resulted in different rates of drying for species with large differences in plant size and hence transpiration rate (Marchin, unpublished; **Figure 3**). Although a difference in drying rate may not be appropriate for all studies, it was acceptable for our primary aim, which was to establish a constant water deficit treatment that could be contrasted to the well-watered control treatment in a large number of species and replicates.

There are several advantages of our simple water deficit method that are ideal for ecological studies. Perhaps most importantly, it requires minimal maintenance throughout the experiment and is relatively inexpensive to implement. Further, the method does not require electricity, wiring, or application of chemicals, and as such, it can be applied inside growth chambers if necessary. We have established that pure sand is not a vital requirement, suggesting that any potting media or soil type could be adapted for use with this method. We have also shown that control plants can be watered *via* drip irrigation, rather than by capillary irrigation inside plastic tubs (**Figure 4B**), which further cuts the materials cost and space requirements. With only a few manipulations of the adjustable water table, it is possible to establish a broad range of soil water deficits of differing intensity, duration, and pulsation (**Figure 2**). For example, mild water deficits could be established by using a lower water table depth (e.g., ≤10 cm), whereas severe water deficits require a greater depth (e.g., ≥25 cm; **Figure 2A**). It is straightforward to simulate gradual droughts of long duration (weeks or months; **Figure 2B**), since daily maintenance consists of simply tracking and adjusting water depths inside plastic drought tubs. Repeated wetting and drying cycles (**Figure 2C**) can also be simulated simply by raising and lowering the water table following the desired schedule. We have also shown that it is possible to incorporate interacting stress factors, such as a heatwave, into the experimental design. Multi-factorial experiments are needed in ecological and physiological investigations, as the importance of determining the interaction of environmental factors is increasingly recognized as critical for our understanding of the impacts of global change (Dermody, 2006).

It is important to consider the desired plant physiological responses before selecting an experimental water deficit treatment, although responses to the same treatment can often differ among species with different water-use strategies (**Figures 7** and **8**). The simulated drought treatment described here resulted in a universal decrease in g_s_ (**Figure 6A**) across a broad range of species with different drought tolerances (**Table 1**). Stomatal conductance of droughted plants was 2–17 times less than that of control plants (**Figure 6A**). Therefore, this same treatment could feasibly be applied to any study aiming to examine plant responses resulting from differences in transpiration rates. For example, studies aimed at understanding plant-insect interactions during drought could use this same moderate water deficit treatment. Our experimental treatment did not, however, decrease Ψ_mid_ for all study species (**Figure 7B**). The most drought-tolerant species, *Grevillea baileyana* and *Buckinghamia celsissima*, maintained Ψ_mid_ at or above their π_tlp_ in control and droughted plants, at least before the heatwave. If drought-related changes in Ψ_leaf_ are an important experimental aim, the method can be easily adjusted to impose a greater intensity and/or longer duration water deficit than was used in these experiments. For example, studies aimed at understanding the physiological processes that occur during drought mortality would require a longer and/or more severe water deficit. While some species wilted and/or dropped leaves during our simulated drought, no plant died during the moderate water deficit until after heatwave temperatures were applied. Therefore, our moderate water deficit can be easily applied to bring plants very close to their physiological limits while ensuring low mortality rates (if desired).

### Simulating Drought and Heat Stress Interactions

The experimental water deficit treatment led to some coherent plant physiological responses. All species within a subset of 11 evergreen trees/shrubs partially closed their stomata in response to simulated drought (**Figure 6A**), resulting in higher T_leaf_ for droughted plants (**Figure 6B**). Stomatal conductance and transpiration result in evaporative cooling of leaves (Nobel, 1974; Farquhar and Sharkey, 1982), so partial stomatal closure under water deficit can result in higher T_leaf_ (Medina and Gilbert, 2015). Transpiration alone can cool leaves by at least 2°C–3°C (Lin et al., 2017) and up to 8°C for species with high transpiration rates (e.g., *M. grandiflora*, **Figure 6**). About half of the species (5 out of 11) experienced declines in Ψ_mid_ that exceeded their π_tlp_ (**Figure 7B**), despite partial stomatal closure (**Figure 6A**). Stomata respond to variations in leaf (or guard cell) water potential (Peak and Mott, 2011; Buckley, 2016) and close before reaching thresholds for xylem cavitation (Bartlett et al., 2016; Martin-StPaul et al., 2017), thus limiting tissue damage under water deficit. The intensity and duration of our experimental treatment led to wilting and/or leaf loss for some, but not all, study species (Marchin, unpublished), indicating the simulated drought stress was moderate overall.

Osmotic adjustment was not observed for any species in the fourth week of water deficit (**Figure 8A**). Some plant species may not be capable of osmotic adjustment, but it is a widely prevalent response to drought, with only 15% of measured species showing no seasonal adjustment of π_o_ (Bartlett et al., 2014). There are two mechanisms for osmotic adjustment of plant leaves: (1) accumulation of organic solutes (e.g., glycine betaine, proline, sugars) and (2) increasing inorganic ion concentrations (e.g., K^+^, Ca^2+^; Chen and Jiang, 2010). Crop varieties with a high capacity for osmotic adjustment can acclimate to drought stress within 6–15 days (Blum et al., 1980; Molinari et al., 2004; Blum, 2017), but our results suggest osmotic adjustment requires a longer response time (i.e., months) in horticultural and wild species.

Interestingly, rapid osmotic adjustment was observed for five species when water deficit was combined with heat stress (**Figure 8B**). This osmotic adjustment occurred in about one week, between the fourth and fifth weeks of water deficit, after 5 days of heatwave temperatures. The importance of π_o_ for high thermal tolerance was first noted for plants growing in extreme environments, such as the deserts of Western Australia (Hellmuth, 1971) and Death Valley, USA (Seemann et al., 1986). Increases in cell sugar concentrations and the resulting decrease in π_o_ may provide a mechanism for rapid temperature acclimation in water-stressed leaves (Santarius, 1973; Huve et al., 2006). In our glasshouse experiments, decreases in π_o_ were observed for trees/shrubs that naturally occur in diverse environments, including temperate forests, tropical rainforests, and sclerophyll forests. These results suggest rapid osmotic adjustment may be a widespread mechanism for plant tolerance of the combination of drought and heat stress. Despite the decrease in π_o_, however, four *H. salicifolia* plants and one *T. erythrocarpum* plant died as a result of drought and heat stress. *Hakea salicifolia* had relatively low wood density and the lowest Ψ_mid_ during the heatwave (**Table 1**), so it is possible that xylem embolism occurred and restricted access to water even after the drought and heatwave ended. Hydraulic failure is a key mechanism leading to tree mortality during drought (Hoffmann et al., 2011; Anderegg et al., 2016), but further research is required to determine why species such as *H. salicifolia* may be particularly vulnerable. The combination of heat and drought stress can quickly kill large swathes of trees (Allen et al., 2010; Williams et al., 2012; Choat et al., 2018), but our current understanding of the physiological mechanisms preceding drought-induced tree mortality is incomplete. Our results indicate that droughted plants are at greater risk of thermal damage (**Figures 6B** and **7B**) and provide valuable insights into how plants cope with multiple stresses (**Figure 8**).

## Conclusions

We have described a simple method for simulating drought effects on plants in glasshouse experiments. Our approach can be easily applied to investigate drought responses: (1) of large numbers of species, provenances, genotypes, etc., (2) to different intensities of a constant stress (mild, moderate, severe), (3) to gradual water deficits of long duration (i.e., months), and (4) combined with interacting abiotic or biotic factors (e.g., heatwaves, atmospheric CO_2_ concentration, nutrient level, mycorrhizal symbiosis, insect pest or pathogen presence, etc.). One limitation to the method is that it resulted in different rates of drying for species with large differences in leaf area and transpiration rate, which may not be appropriate for all studies. It is also important to note that the method was tested in a highly-controlled glasshouse, and variations or fluctuations of soil drying profiles are to be expected when attempting to use this method in less-controlled environments, where, for instance, changes in PAR or VPD can affect plant evapotranspiration and thus drying rates. As demonstrated here, this method can be used to rank species according to drought tolerance and elucidate species’ differences in physiological mechanisms for coping with drought and heat stress. The most drought-tolerant species in our study was *Buckinghamia celsissima* (π_tlp_ = ‒2.3 MPa), based on the π_tlp_ ranking, whereas *Castanospermum australe* (π_tlp_ = ‒1.7 MPa) is sensitive to drought and should be avoided for urban plantings in drought-prone cities with frequent water limitations or imposed restrictions. The most vulnerable species to drought in combination with heat stress was *Hakea salicifolia* (π_tlp_ = ‒2.1 MPa), indicating that the interactive effects of heat and drought stress are complex and cannot easily be predicted based on measurement of π_tlp_ alone. We recommend this simple water deficit method for the study of plant drought responses in a range of ecological contexts, particularly those involving other abiotic or biotic effects.

## Data Availability Statement

The raw data supporting the conclusions of this article will be made available by the authors, without undue reservation, to any qualified researcher.

## Author Contributions

ML and DE conceived the ideas and the overall study design. RM and AO designed the methodology and collected the data. RM analysed the data. RM led the writing of the manuscript. All authors contributed critically to the drafts and gave final approval for publication.

## Funding

This is a contribution from the Which Plant Where project, which is funded by the Green Cities Fund, as part of the Hort Frontiers Strategic Partnership Initiative developed by Hort Innovation Australia, with co-investment from Macquarie University, Western Sydney University, and the NSW Department of Planning, Industry and Environment, and funds from the Australian Government.

## Conflict of Interest

The authors declare that the research was conducted in the absence of any commercial or financial relationships that could be construed as a potential conflict of interest.
