# Supplementary material for: A Simple Method for Simulating Drought Effects on Plants
Source: Front Plant Sci. 2020 Jan 21;10:1715. doi: 10.3389/fpls.2019.01715 (PMC6985571; doi:10.3389/fpls.2019.01715)
Supplement: Supplementary file 1 [file DataSheet_1.pdf]

Supplementary Material

1 Supplementary Tables

SUPPLEMENTARY TABLE S1 | List of 50 plant species/varieties exposed to water deficit treatment in one of two glasshouse experiments. The family, growth form, and origin (relative to Australia) are described for each species.

Species/Horticultural variety	Family	Growth Form	Origin
Hardenbergia violacea (Schneev.) Stearn	Fabaceae	Climber/Groundcover	Native
Hibbertia scandens (Willd.) Dryand.	Dilleniaceae	Climber/Groundcover	Native
Kennedia beckxiana F.Muell.	Fabaceae	Climber	Native
Kennedia prostrata R.Br.	Fabaceae	Groundcover	Native
Myoporum parvifolium R.Br., var. Purpurea	Scrophulariaceae	Groundcover	Native
Pandorea jasminoides (Lindl.) K.Schum., var. Jazzy Bellz	Bignoniaceae	Climber	Native
Trachelospermum asiaticum (Siebold & Zucc.) Nakai	Apocynaceae	Climber/Groundcover	Exotic
Cordyline australis (G.Forst.) Endl., var. Red Sensation	Asparagaceae	Herb	Exotic
Dianella caerulea Sims, var. Goddess	Xanthorrhoeaceae	Herb	Native
Liriope muscari (Decne.) L.H.Bailey	Asparagaceae	Grass	Exotic
Lomandra longifolia Labill., var. Verday	Asparagaceae	Grass	Native
Stenotaphrum secundatum (Walter) Kuntze, var. Sapphire	Poaceae	Turf	Exotic
Plectranthus argentatus S.T.Blake	Lamiaceae	Herb	Native
Zoysia macrantha Desv., var. Nara	Poaceae	Turf	Native
Acacia implexa Benth.	Fabaceae	Tree	Native
Agonis flexuosa (Muhl. ex Willd.) Sweet, var. Burgundy	Myrtaceae	Shrub	Native
Alectryon coriaceus (Benth.) Radlk.	Sapindaceae	Tree	Native
Alectryon oleifolius (Desf.) Reynolds	Sapindaceae	Tree	Native
Atractocarpus fitzalanii (F.Muell.) Puttock	Rubiaceae	Tree	Native
Backhousia citriodora F.Muell.	Myrtaceae	Shrub	Native
Backhousia myrtifolia Hook. & Harv.	Myrtaceae	Shrub	Native
Buckinghamia celsissima F.Muell.	Proteaceae	Tree	Native
Castanospermum australe A.Cunn. & C.Fraser	Fabaceae	Tree	Native
Correa sp., var. Catie Bec	Rutaceae	Shrub	Native
Cryptocarya laevigata Blume	Lauraceae	Tree	Native
Cryptocarya mackinnoniana F.Muell.	Lauraceae	Tree	Native
Cupaniopsis anacardioides (A.Rich.) Radlk.	Sapindaceae	Tree	Native
Delonix regia (Hook.) Raf.	Fabaceae	Tree	Exotic
Dysoxylum fraserianum (A.Juss.) Benth.	Meliaceae	Tree	Native
Ficus microcarpa L.f., var. Hillii	Moraceae	Shrub	Native
Flindersia australis R.Br.	Rutaceae	Tree	Native
Flindersia maculosa (Lindl.) Benth.	Rutaceae	Tree	Native
Grevillea baileyana McGill.	Proteaceae	Tree	Native

SUPPLEMENTARY TABLE S1, cont.

Species/Horticultural variety	Family	Growth Form	Origin
Hakea laurina R.Br.	Proteaceae	Shrub	Native
Hakea salicifolia (Vent.) B.L.Burtt	Proteaceae	Shrub	Native
Harpullia pendula Planch. ex F.Muell.	Sapindaceae	Tree	Native
Hymenosporum flavum F.Muell.	Pittosporaceae	Tree	Native
Lophostemon confertus (R.Br.) Peter G.Wilson & J.T.Waterh.	Myrtaceae	Tree	Native
Magnolia grandiflora L., var. Little Gem	Magnoliaceae	Tree	Exotic
Melastoma affine D.Don	Melastomataceae	Shrub	Native
Murraya paniculata (L.) Jack	Rutaceae	Shrub	Exotic
Pittosporum tobira (Thunb.) W.T.Aiton, var. Miss Muffet	Pittosporaceae	Shrub	Exotic
Sarcopteryx stipata (F.Muell.) Radlk.	Sapindaceae	Tree	Native
Syzygium floribundum F.Muell.	Myrtaceae	Tree	Native
Syzygium luehmannii (F.Muell.) L.A.S.Johnson	Myrtaceae	Tree	Native
Syzygium wilsonii (F.Muell.) B.Hyland	Myrtaceae	Shrub	Native
Toechima erythrocarpum (F.Muell.) Radlk.	Sapindaceae	Tree	Native
Tristaniopsis laurina (Sm.) Peter G.Wilson & J.T.Waterh.	Myrtaceae	Tree	Native
Tristaniopsis laurina, var. Luscious	Myrtaceae	Tree	Native
Xanthostemon chrysanthus (F.Muell.) Benth.	Myrtaceae	Tree	Native

SUPPLEMENTARY TABLE S2 | Mean osmotic potential at full turgor (π_o, MPa), water potential at turgor loss point (π_{tlp}, MPa), relative water content at turgor loss point (RWC_{tlp}, percent), and bulk modulus of elasticity (ε, MPa) ± SE for 27 tree/shrub plus 2 liana species. Data was extracted from pressure-volume curves of leaves from well-watered plants (*n*=1–10 leaves per species).

Species/Horticultural variety	*n*	π_o (MPa)	π_{tlp} (MPa)	RWC_{tlp} (%)	ε (MPa)
Alectryon coriaceus	10	-1.60 ± 0.06	-1.83 ± 0.06	90.42 ± 1.00	17.42 ± 1.99
Alectryon oleifolius	1	-2.28	-2.33	94.73	34.23
Atractocarpus fitzalanii	1	-1.76	-1.89	94.11	11.55
Backhousia citriodora	7	-1.65 ± 0.08	-1.93 ± 0.05	89.17 ± 1.84	15.55 ± 1.63
Backhousia myrtifolia	2	-1.48 ± 0.51	-1.59 ± 0.52	93.28 ± 0.41	11.24 ± 2.38
Buckinghamia celsissima	3	-2.26 ± 0.08	-2.47 ± 0.09	94.01 ± 0.36	10.37 ± 0.88
Callistemon 'King Park Special'	2	-2.62 ± 0.18	-2.88 ± 0.15	94.11 ± 0.26	9.41 ± 1.26
Castanospermum australe	4	-1.14 ± 0.06	-1.36 ± 0.03	93.67 ± 0.10	6.16 ± 1.08
Ceratopetalum apetalum	4	-1.21 ± 0.09	-1.50 ± 0.04	89.30 ± 1.17	13.14 ± 0.78
Corymbia citriodora	1	-2.24	-2.49	93.46	8.53
Cryptocarya laevigata	2	-1.90 ± 0.27	-2.04 ± 0.34	93.88 ± 0.60	15.18 ± 5.15
Cupaniopsis anacardioides	2	-1.42 ± 0.06	-1.58 ± 0.04	93.96 ± 0.12	8.51 ± 0.89
Dysoxylum fraserianum	10	-1.66 ± 0.06	-2.06 ± 0.04	87.98 ± 0.97	16.30 ± 2.53
Ficus brachypoda	1	-1.54	-1.64	93.37	15.00
Ficus microcarpa	4	-1.31 ± 0.08	-1.49 ± 0.08	92.91 ± 1.35	15.61 ± 3.22
Flindersia australis	10	-1.34 ± 0.09	-1.80 ± 0.04	89.57 ± 1.00	13.00 ± 1.53
Grevillea baileyana	1	-2.24	-2.49	93.52	8.58
Hakea laurina	1	-2.00	-2.14	93.94	13.25
Hardenbergia violacea	6	-1.22 ± 0.13	-1.28 ± 0.13	93.65 ± 0.59	36.58 ± 10.00
Harpullia pendula	4	-1.93 ± 0.07	-2.02 ± 0.09	93.08 ± 0.54	21.70 ± 4.01
Kennedia beckxiana	4	-1.11 ± 0.04	-1.22 ± 0.05	93.78 ± 0.24	11.03 ± 2.26
Magnolia grandiflora	4	-1.31 ± 0.02	-1.44 ± 0.03	93.21 ± 0.14	9.30 ± 0.75
Syzygium floribundum	1	-1.16	-1.30	93.84	7.66
Syzygium luehmannii	6	-1.27 ± 0.11	-1.73 ± 0.15	86.02 ± 2.42	5.95 ± 1.11
Syzygium wilsonii	1	-2.18	-2.23	94.28	32.85
Toechima erythrocarpum	3	-2.21 ± 0.02	-2.33 ± 0.03	94.93 ± 0.28	18.41 ± 1.96
Tristaniopsis laurina	4	-1.84 ± 0.11	-2.16 ± 0.06	86.43 ± 2.43	9.32 ± 2.64
Xanthostemon chrysanthus	1	-1.20	-1.34	94.21	7.98
Xanthostemon paradoxus	1	-1.29	-1.37	93.85	14.81

2 Supplementary Figures

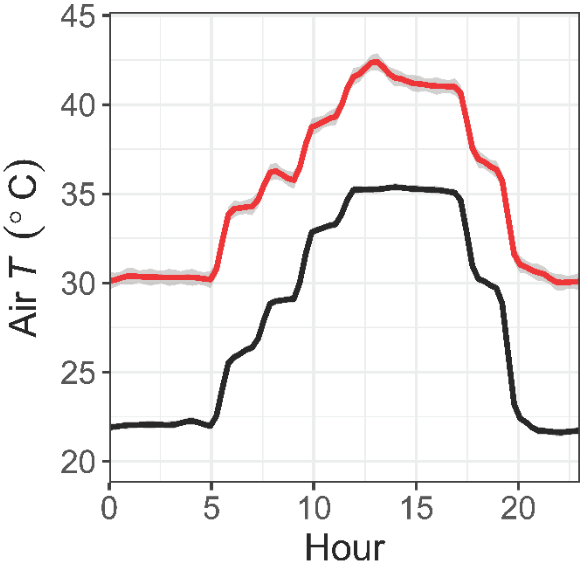

SUPPLEMENTARY FIGURE S1 | Air temperature (°C) inside the glasshouse during the water deficit phase (black line) and heatwave phase (red line) of the HIE experiment on an austral summer day (14 February 2018). Daily mean temperature was 27 °C to represent summer conditions in southeastern Australia, with a diurnal range from 21 to 34 °C and the maximum temperature spanning six hours at midday (12:00–18:00). Mean heatwave temperature was 35 °C, with a diurnal range of 30–41 °C and the maximum temperature spanning two hours at midday (12:00–14:00).

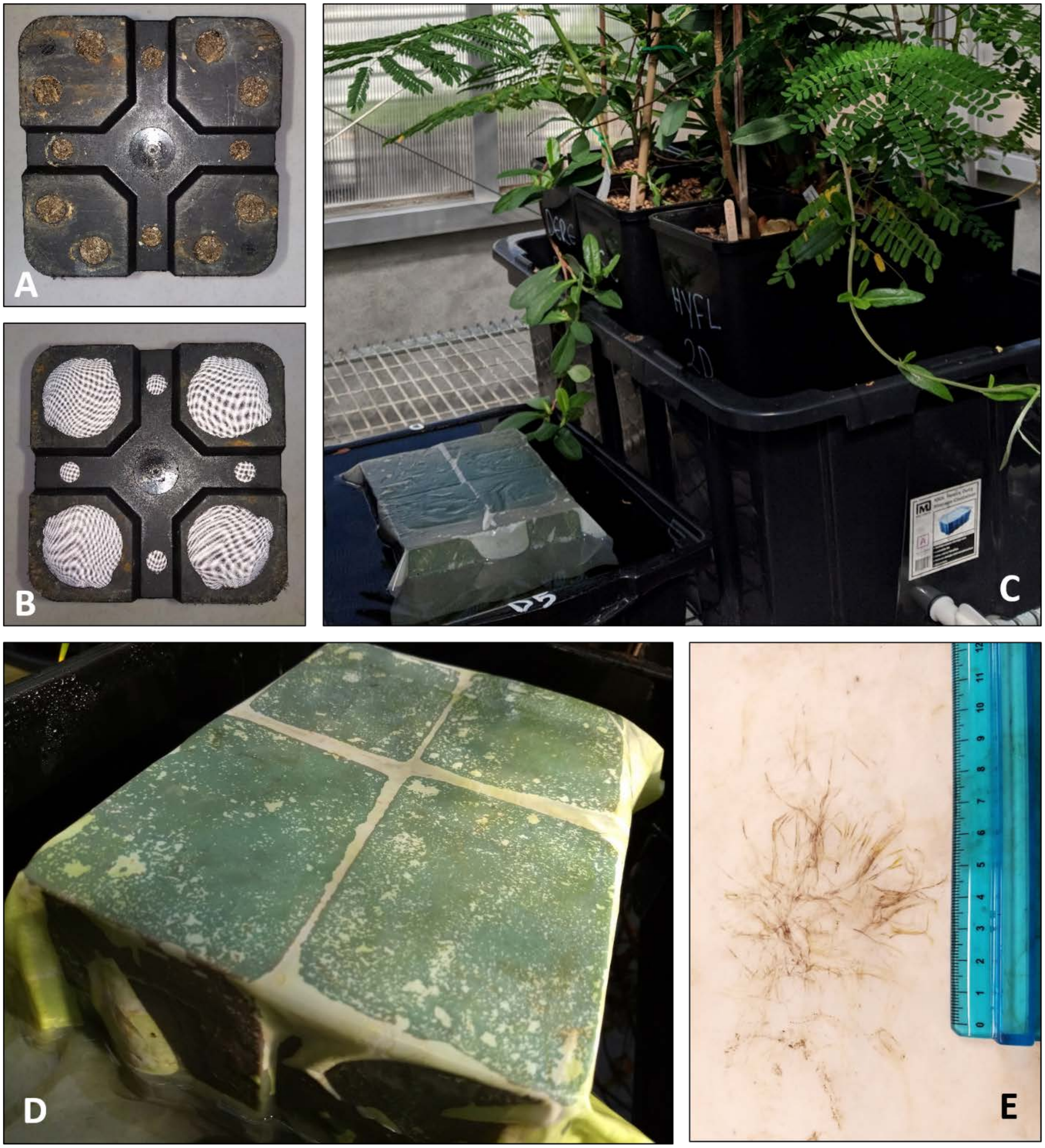

SUPPLEMENTARY FIGURE S2 | (A) The flat base of a control pot used with drip irrigation. **(B)** The flat base of a drought pot containing four large 4.3-cm diameter circles, which ensured adequate contact area between the soil at the bottom of the pot and the foam surface for capillary irrigation. **(C)** A 23-cm column of commercial porous foam (lower left, Oasis IDEAL Floral Foam Maxlife brick; Smithers-Oasis, Kent, OH, USA) fitted with fine nylon mesh (20-µm, Allied Filter Fabrics, Berkeley Vale, NSW). Potted plants are placed on top of foam (see **FIGURE 1**). **(D)** Detail of the foam surface rewetted after two rounds of trial experiments. Surface wetting capacity is still largely retained, thus allowing reuse for new drought experiments. **(E)** Detail of the root barrier after a pot was left to overgrow on the foam surface. The nylon mesh allowed exchange of air and water but prevented root passage into the foam.

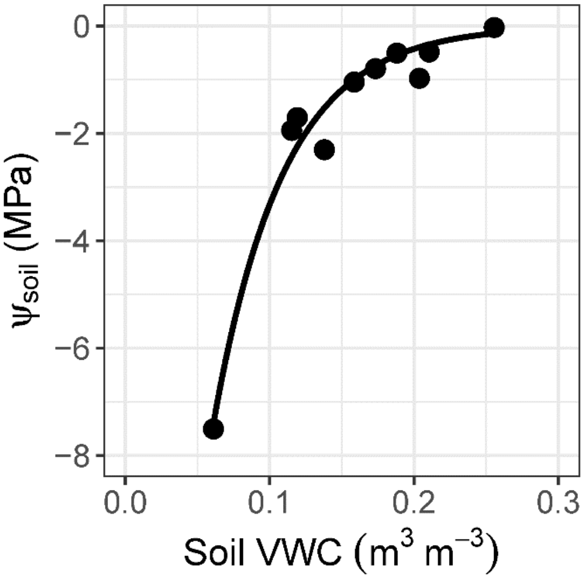

SUPPLEMENTARY FIGURE S3 | The soil moisture characteristic curve for the potting mix used in the glasshouse experiments. Soil volumetric water content (VWC) at field capacity (Ψ_{soil} = –0.01 MPa) is ~35%, whereas soil VWC at permanent wilting point (Ψ_{soil} = –1.5 MPa) is 14%.
